# Mr. Ring's Case of Tic Douloureux

**Published:** 1807-08

**Authors:** John Ring

**Affiliations:** New-Street, Hanover-Square


### *To the Editors of the Medical and Physical Journal.*

Gentlemen,

I Communicate the following Case of Tic Douloureux, in hopes that the treatment pursued on this occasion may prove equally successful in other similar cases. The cicuta, which so readily succeeded in this instance, as well as in others, although frequently administered in such complaints, is sometimes given too sparingly to effect a cure.

Mr. Shoubridge, of Old Bond Street, was troubled with a painful affection of the face twelve or fourteen years.—The disorder has been rather hereditary in his family; which induced him almost to despair of finding a remedy. At first he attributed it to his teeth; in consequence of which, he submitted to the extraction of all those which were decayed, but without obtaining any permanent relief.

For the space of two years previous to his applying to me on this account, he had generally the most excruciating torture after every meal; insomuch as sometimes to make him faint, and fall out of his chair. It occasionally lasted an hour or two, attended with alternate exacerbations and remissions, like a spasmodic disease. When the pain was most violent, it was of the lancinating kind.

A few months before, he had been under my care, in consequence of his rupturing the tendon of the rectus muscle; and the necessary confinement to his bed, by depriving him of the advantage of exercise, aggravated his complaint.

He had tried various remedies, but in vain.—I first gave him cicuta in small doses; directing him to increase them gradually; but not receiving any immediate benefit, he soon discontinued its use. He had heard of cures performed in such maladies, by the division of the nerves; and was anxious to have such an operation performed; but as the pain was in the lower jaw, I told him it was impracticable to perform it, with any hope of success. He was, however, impatient; and, with my consent, consulted an eminent surgeon, who coincided in opinion with me.

This gentleman advised him to take ten drops of the arsenical solution every six hours; increasing the dose gradually to fifteen; but in the course of a few days, his bowels were so much disordered by ten drops, that I advised him to discontinue it. He then promised to give a fair trial to any medicine which I should recommend. I accordingly gave him the cicuta; directing him to take two pills every morning and noon, and four at bed-time; and to increase the dose as far as he could conveniently bear.

This plan had the desired effect. Within a few days his pain was much alleviated; and in a fortnight perfectly removed. More than two years have now elapsed; yet he has never had the slightest return of it, from that period to the present hour.

I have known this medicine prove equally efficacious in other instances; but only one in which the complaint was so violent, as in that of Mr. Shoubridge. In his case, the cicuta was increased to five scruples a-day; in the other to seven.

---

Since the preceding case was drawn up, your Journal for June has appeared; in which are some errors of the press, which I beg the favour of you to correct. P. 543, l. 16, for began, r. begun. Table of contents, and superscription of p. 540, &c. for Mr. Anderson, r. Dr. Anderson.

These are not the only errors committed in the last Number of your Journal. A false alarm is there sounded by a Mr. Golding, of Reading; and I rather wonder, that the vigilance of the editors permitted it to pass unnoticed. This gentleman is an additional proof of the justice of Dr. Jenner's observation, that *it is surprising how many people write on this subject; and how few understand it.*

Mr. Golding says, he by no means *wishes* to be considered as an anti-vaccinist; and indeed, it is no very desirable ble character; when we consider, with what sort of persons he would be ranked, if he were to be considered in that light.

Mr. Golding says, the cow-pock in all the five patients whom he vaccinated, went through the regular stages; yet he afterwards tells us, he has this circumstance to mention, concerning one of them, namely, that of Eliza, "after the disease had gone through its regular stages, and the habit had been affected, the part where the matter had been inserted, degenerated into what Dr. Jenner calls erysipelatous inflammation; the wound became deeper and larger, and a secondary constitutional affection was produced. Hence it appears, that the disease did not go through its regular progress, as Mr. Golding asserts; but either was interrupted in its course, or was never of the genuine kind.

Mr. Golding says, he has never heard or read of the secondary indisposition destroying the previous constitutional affection. So absurd an hypothesis, as that of a subsequent disorder destroying the security imparted by the preceding, it is probable never entered the mind of but one man. Had Mr. Golding, however, heard any thing, or read any thing, about the cow-pock, it is probable he would have known, that many instances of the small-pox, either in a perfect or a partial degree, have occurred, in those cases where the cow-pock has been interrupted in its progress, and that nothing less is required by its advocates, than that the process of exsiccation and scabbing, as well as that of vesication, should be regular and complete. In proof of this, the Jennerian Society, as well as other acknowledged judges of the practice, require, that where there is any considerable deviation from the ordinary course of the cow-pock, the patient should be vaccinated again.

As to the formidable train of symptoms with which Eliza was affected, it is totally unnecessary to say a single word more about them; since Mr. Golding himself is of opinion, that they proceeded from the quinsey, and not from the small-pox. Charity, therefore, must induce us to suppose, that although these formidable symptoms in Eliza, add a little shade to the picture, they were pourtrayed, only to maintain the fidelity of the narrative, and not for the sake of blackening the character of vaccination.

It is much to be regretted, that Mr. Golding did not revaccinate this subject, either after the cessation of the erysipelatous affection, or previously to his putting her to the test test of variolous inoculation. Even those who, after the promulgation of Mr. Goldson's opinion, propagated the loudest alarm, now allow, that the vaccine test is as efficacious as the variolous; and we all know that it is much more safe. At any rate, no persons should be subjected to the test of variolation, till they are armed with all the security which vaccination can afford.

The more we scrutinize Mr. Golding's communication, the more we discover, how utterly unacquainted he is with that branch of the healing art, on which he has thought proper to take up his pen. You have expressed your intention of discontinuing, or at least abridging, discussions of this kind; but from the specimen now before us, of persevering ignorance, after what has been done to inform the public mind, you may possibly be induced to consider such a resolution premature.

As one instance of Mr. Golding's ignorance of this subject, in addition to the former, he takes care to let us know, that a local pustule was excited by the variolous test in another of his patients; but he does not guard his readers from error, by informing them, that a similar pustule may in some instances be excited, in a person who has had the small-pox. Dr. Valentin, Mr. Fewster, and many other authors, relate cases of this sort; in which matter taken from such local pustules, in the secondary small-pox, has infected others by inoculation, and communicated the genuine disease.

Dr. Jenner mentions an instance of the kind, on the authority of a late professor at Edinburgh; in which a great inoculator for the small-pox used to carry the matter for infection in his own arm. This anecdote has been confirmed to me by Mr. Jones, an eminent medical practitioner, of Mount Street; who tells me, that the inoculator here alluded to was Mr. Thomas Sutton; and that he heard the same anecdote from his own mouth.

Mr. Kite, late of Gravesend, informed me, that he had inoculated himself for the small-pox, many years after he had had the disorder; and produced matter, which he had sent to Dr. Mitchell of Chatham; and that this matter had proved effectual in subsequent inoculations. I trust, therefore, we shall hear no more of such inoculations; which, whether they are intended to spread an alarm or not, have frequently had that effect, particularly in the Portsmouth cases, the Woolwich cases, and the Harrow cases. These have have all, in their turn, served as so many bugbears, to frighten the public out of their senses, and to deter them from vaccination; and have all been exploded. The local variolous pustule, when excited after vaccination, has therefore had its day; and can no longer impose upon the intelligent part of the community; but it may certainly still prove sufficient to spread a false alarm, and create a popular delusion.

---

I now beg leave to refute some other cases, brought forward by Dr. Rowley.—The 146th case, in the 2nd edition of his book, is that of Mr. Perceval. The Doctor tells us, that this gentleman is *now living at Gosport*; that he was vaccinated at the Small-pox Hospital four years ago, and *died of the small-pox the same year.*

Nos. 186 and 187, the child of John Lea, and that of John Mason, vaccinated by me. Dr. Rowley says these children had the small-pox. Dr. Willan, on the contrary, in a letter now in my possession, declares, that it was the chicken-pox; and that if he ever saw it more distinctly marked in one instance than in any other, it was in the present. Many respectable practitioners were of the same opinion, particularly Dr. Hooper.

No. 192, the child of Mr. Guinigo. Dr. Rowley says this child was vaccinated by me. This is a mistake; and furnishes one instance of the facility with which Dr. Rowley, and the other enemies of vaccination have admitted reports unfavourable to the practice. Mrs. Curling, of George Street, Portland Chapel, whose child was the subject of another erroneous report, acknowledged to me, that she had communicated this case to Dr. Rowley, in return for some supposed professional services. Dr. Rowley recorded it, as reported by Mrs. Curling, without inquiring into it.

The fact is, that the child was vaccinated, not by me, but by Dr. Thornton, about three years before the occurrence of the pustules which gave rise to this report. A younger child then had the confluent small-pox; and the other, who was dropsical, and perfectly helpless, lay on the same bed. The mother attended them, and alternately handled them; in consequence of which, the child in question had eleven pustules. His mother was of opinion, that he was rather more indisposed at the time of their appearance.

pearance. This is not improbable; but it may reasonably be doubted whether this was a consequence, or a mere coincidence, as she confesses he had been given over some time.

A very partial and unfair representation has been given of the case of Mrs. Curling's own child, by Dr. Moseley and Dr. Rowley. Dr. Moseley pretended, that the child had had the small-pox after vaccination. In my pamphlet in answer to him, I proved that this charge was totally destitute of foundation. Mr. Merriman also proved the same, both in his pamphlet and in the Gentleman's Magazine; nevertheless, Dr. Rowley afterwards inserted the case in his list of failures, as if it had never been refuted. This is the way in which the antivaccinists multiply their cases. Another is by relating the same case twice; of which, many instances may be found in Dr. Rowley's publication.

Dr. Moseley says, Curling's child had ulcerations about his body, and was otherwise much disordered, *after* the cow-pox; and Dr. Rowley says, he was much diseased with eruptions and ulcers *after* cow-pox. This is a very serious charge against the cow-pox: it is possible, however, that these gentlemen, and their friends, have had ulcerations about their bodies, and been much diseased, *after* the small-pox, without any imputation on that disorder.

As this case has made a great noise, and caused considerable uneasiness in the minds of many anxious parents, I have once more inquired into it; and flatter myself, neither you nor your readers will have any objection to hearing the result.—Three weeks after the birth of this child, Mrs. Curling caught cold; and had a pain in her head, with which she has been troubled at times ever since. This had aggravated a nervous affection, under which she previously laboured. The child was vaccinated when ten months old; and weaned a few days after. From this period his health began to decline; which is no great wonder, when we consider that he was deprived of his best support at such a critical age. Six months after this, he had an eruption about his mouth, which continued three weeks. This is no more than what frequently happens to children, either from cold or teething; and would not have been thought detrimental, had it not happened *after* the cow-pox.

About nine months after vaccination, the disease occurred, which Dr. Mosely stated to be the small-pox, and declared *there could be no doubt of it;* though he had *never*

seen

*seen it*, and though the mother told him she did not know it to be the small-pox; and that the only two medical men who had seen it, pronounced it to be the chicken-pox.—So ready are the most sceptical of the antivaccinists, to believe what they wish to be true.

It was not till after this last disorder, that the child had the eruption which gave his mother the most concern. This was on the head; and whether occasioned by the small-pox or the chicken-pox, there is every reason to believe, would never have happened, but from want of care. It is no uncommon circumstance to meet with the tinea capitis after the chicken-pox; from a neglect of cutting off the hair round the sores. The accuracy of the statement here given may be relied on; since it was given to me by Mrs. Curling herself, and signed with her own hand.

The 101st case related by Dr. Rowley, is that of the child at Clapham, in whose arms, he declares, the matter produced the desired effect.—In my Treatise on the cow-pox, I have proved the contrary. He says, on the ninth day a spreading inflammation covered the body. This is not true. The inflammation, which was occasioned by the injury done to the arm, and not by inoculation, only commenced about that time, and gradually spread in consequence of neglect. Many days elapsed, before it had spread over the whole body; and there is every reason to conclude it would never have spread at all, had proper care been taken. The remark which Dr. Rowley makes on this case is curious. He says, " The matter supposed to be taken too late, which is candidly granted, and *shews vaccine malignity*."—How this is reconcileable to reason, is left to the *antivaccine malignity* of his surviving fellow-labourers to explain.

The 242nd case related by Dr. Rowley is that of a child of Mr. Richardson, in Cleveland Street. Dr. Rowley says, this child was buried by Mr. Carlisle of Clipstone Street; and it would be happy if we could bury the falsehood which he has brought forward on this occasion in the same grave. But justice to the present age and to posterity demands, that such a daring violation of truth should be exposed and corrected.

Dr. Rowley, in his zeal to destroy the credit of vaccination, not only represents this child to have died of the small-pox after the cow-pox; but to have died of the small-pox, after the cow-poxers had insured security. I have therefore inquired into the case; and am informed by Mrs. Richardson, that the child was inoculated for the cow-pock cow-pock by Mr. Jones, then of Wigmore Street, but infection did not take place; and as Mrs. Richardson was then far advanced in her pregnancy, she proposed to defer inoculating it again till after her delivery; when she meant to have the two children inoculated at the same time. This, however, was prevented by the occurrence of the natural small-pox; which proved fatal. Here we see one instance of the base fabrications brought forward by certain medical men, who are interested in the question, to prejudice the public.

The 34th case brought forward by Dr. Rowley, is that of Miss Lutwidge, who is also stated by him to have had the small-pox after the cow-pox. This is the more extraordinary, when Dr. Rowley himself tells us, that the very gentleman who inoculated her, says she was not inoculated with the cow-pox but with the small-pox.

I have now before me a letter from Mr. Bliss, surgeon, of Hampstead, the practitioner alluded to, whom Dr. Rowley calls by way of reproach the gentleman vaccinator, positively declaring, that he inoculated Miss Lutwidge with the small-pox, and not with the cow-pock; as a confirmation of which, it was done in the year 1797, before Dr. Jenner's publication appeared. She had an eruption of pustules; and three years after, had a full eruption of the small-pox.

Mr. Bliss inoculated a girl in 1793; who likewise had several pustules, and considerable constitutional indisposition. She was afterwards three times exposed to variolous infection, in her employment of nursing children in that disorder. In the year 1798, however, she had a full crop of the natural small-pox; with much constitutional affection.

The 138th case brought forward by Dr. Rowley, is that of Mr. Twyford's daughter, of Wilsdon Green; whom he also states to have been inoculated for the cow-pock. He acknowledges, however, that this point was disputed. It is therefore evident, that had he not been determined to press every unfavourable report into the service of the antivaccinists, whether true or false, he would either have omitted this case; or have endeavoured to substantiate it by some kind of evidence.

I have now before me the copy of a letter from Mr. Haines, surgeon, of Hampstead, partner of Mr. Bliss, transmitted, with the former communication from Mr. Bliss to the Jennerian Society, in which he proves the gross and palpable falsity of this case also. Dr. Rowley says, says, this girl had a very thick small-pox after vaccination. Mr. Haines being at Wilsdon Green, and knowing the case of Miss Lutwidge, related by Dr. Rowley, to be totally destitute of foundation, inquired into this also; and was informed by Mrs. Twyford, that this child and two others had been inoculated for the small-pox six years before, by Mr. Collins of Wilsdon, from a man who caught the small-pox in London, and communicated the infection in the natural way to his own children.

The three children who were inoculated, sickened at the usual time. The girl who is the subject of the present case, was exceedingly ill; and delirious one or two nights. Several pustules appeared round the inoculated part; which rose very finely. The progress of the disease was similar in all the children; and Mr. Collins pronounced them all secure from the future infection of the small-pox.

The girl has since had the small-pox severely. Dr. Rowley never saw her in either of the complaints; nor does it appear, that he ever took any pains to know whether the report was true or not. Having vowed that he would put an end to vaccination, he readily received and published every report that was unfavourable to the practice. When this girl was inoculated, her mother was so far from thinking it was with the cow-pock, that she had never even heard of the disease. I was yesterday at Wilsdon; and this instance of secondary small-pox was related to me by the sister-in-law of the patient; who was an eye-witness of the whole case.

The 418th case related by Dr. Rowley, is that of William Shepherd, said to reside in Chandler Street, in the same house where another case is also represented to have occurred. No number is mentioned; and I have tried to find out in what house William Shepherd lives, but in vain. The first case is reported by Dr. Rowley, on the authority of Dr. Moseley; who is said to have received the information from the mother of the child. Whether Dr. Rowley had Dr. Moseley's authority for the case, in which I am said to have been concerned, I know not; but whatever authority he had, I shall prove by the best authority possible, that the report is totally destitute of foundation.

It is stated in Dr. Rowley's book, that this William Shepherd was vaccinated by me. This is a point which may be ascertained, when the antivaccinists will inform us where he is to be found. It is also stated, that he has since been inoculated for the small-pox, by Mr. Richardson son of Sloane Street; and that he went through the disease in a regular manner.—I have inquired of Mr. Richardson, whether he knows any thing of such a case; and he declares it is an utter falsehood. He never inoculated this William Shepherd; and never knew him.

This is one of the 218 fresh cases, brought forward by Dr. Rowley in the 2nd edition of his work. He tells us, they all came to light within a few days, with little solicitation on his part; which shews how ardently some people were labouring for the public good. He pretends that the friends of vaccination endeavoured to conceal these cases. To me, however, it appears, that those who propagate such reports, only expose their own folly; and those who wish to conceal them, or to prevent them from being thoroughly investigated, are no friends of vaccination.

The preceding case, in Dr. Rowley's book, is that of the daughter of Mr. Thompson, in Chandler Street, said to have been vaccinated by Mr. Leese, and afterwards to have died of the small-pox. This information is said to have been given by her mother to Dr. Moseley. The number of the house, however, where this Mrs. Thompson lives is carefully concealed; and although Mr. Leese has examined his register, he cannot find any such name. I caused an inquiry to be made by my Assistant throughout the Street; but he could not discover the residence of this or the preceding subject; so that we must wait for an investigation of the truth of the story, till Mrs. Thompson comes forward to attest it.

In another of Dr. Rowley's cases, mention is made of an abscess in the side; which is there ascribed to the cow-pox; but I have seen a letter from the medical man who attended the patient, from which it appears, that it was occasioned by a fall, which broke one of the child's ribs. Every true antivaccinist, however, will conclude, that it was all owing to the cow-pox.

When Dr. Rowley wrote his book, such encouragement was given to those who brought unfavourable reports, that it is well known, many of them were mere fabrications.—They were, however, all readily received, speedily published, and eagerly swallowed by the credulous multitude; many of whom have now great reason to repent of their folly. So rapidly, indeed, did these pretended cases of cow-pock failure, and cow-pock disasters, accumulate, that Dr. Rowley tells us, his senses were appalled, and his pen tired of recording them. A considerable number of them them appear to have been nothing but the itch; and when the case proved obstinate, either from neglect, or any other cause, the disease was attributed to vaccination.

I shall here quote one passage from Dr. Rowley's book, as an additional proof, how far he is worthy of credit.— "I have been in some vaccine storms; and have had *the buttons torn off my coat, cloth and all,* to convince me of the great and infallible excellence of cow-pox. I have seen some few of the vehement vaccinators redden like a flame with fury; *the lips quivering, the eyes starting out of the head, with flashing streams of fire;* the mouth foaming, and tongue pouring forth a torrent of hard words, like a thunder storm,—the fist clenched like a pugilist, ready to accompany the violent wrath with *other* knock-down arguments."—We are then given to understand, that the *mild* enemies of vaccination quit the scene; and, indeed, it is no wonder that these *gentle* and *modest* inquirers after truth should quit the scene; and fly from such formidable opponents.

Mr. Blair, in his publication entitled The Vaccine Contest, proved the falsehood of many of Dr. Rowley's statements, from his own book; and observes, that in a great proportion of the cases brought forward by him, he has not mentioned the names or places of abode of the patients; nor by whom they were vaccinated, if they ever were vaccinated at all. He has also alleged, that twelve or thirteen of these subjects caught the cow-pox by milking; without adducing any evidence to justify his assertion: and in no less than one hundred and forty-five cases, the patients appear to have been infected with the small-pox previously to vaccination.

Dr. Rowley is of opinion, that inquiries relative to the particulars, and the reality of the cases brought forward, may *confound fools;* but Mr. Blair justly observes, that none but *fools of the most dangerous stamp* would endeavour to decide in any case, without making such inquiries. He also observes, that the example of Dr. Rowley, in taking these idle reports on trust, has been too often followed; in consequence of which the public mind has been agitated, and grossly misled, respecting vaccination.

Mr. Blair is of opinion, that any person who peruses a fourth part of the works written in favour of the practice, will see the extent of the frauds, as well as the mistakes, of the antivaccinists; and perceive, that many of the cases which they hold up to the public notice, have been refuted again and again. He will perceive, that these adventurers venturers have been raking the very kennels of human misery for diseases, in order to impute them all to vaccination.

After modestly confessing, that his own experience has not been equal to that of many others in this department of medicine, Mr. Blair informs us it has nevertheless been such as to justify him in asserting, that it would be nothing short of insanity to discontinue the practice of vaccination; to substitute the curse of the variolous pestilence in its stead; and to scatter among our peaceful fellow-citizens, in this great metropolis, firebrands and death!

Dr. Rowley having stated, that it is the duty of *honourable men* to alarm mankind, and that in some places people were now inoculated for the small-pox by farriers, at five shillings a head, Mr. Blair observes, that the zeal of *many farriers,* and such like *honourable men,* in spreading a direful pestilence, *for the sake of a little pelf*, is acknowledged and deeply lamented. He then remarks, that the insatiable love of money unhappily prevails; but he is at a loss to conjecture, why this scourge of human nature continues to be purposely inflicted at the Small-pox Hospital, and sent forth to desolate the inhabitants of London. He knows not how to reconcile this conduct with the original design of that Institution, when the Governors declared by their last Report, that the constant success of vaccination "had confirmed all that was promised by so invaluable a discovery."

Dr. Rowley thinks a change of system necessary in the Small-pox Hospital; and Mr. Blair agrees with him. He is decidedly of opinion, that the continuation of Small-pox Inoculation Hospitals in this vast metropolis is a fertile source of misery and death. He wishes them to be set apart entirely and exclusively as *pest-houses,* to receive all those patients who have casually become infected with the small-pox; but never to be employed for the purpose of inoculation. He justly remarks, that while the contrary practice prevails, and is encouraged, we cannot exterminate the small-pox from this populous city; since there will never be wanting sordid and avaricious persons, who, from motives of self-interest, will perpetuate the stock of contagion.

As to the open and professed opponents of vaccination, Mr. Blair observes, that while they reproach the friends of the practice for not accomplishing their humane intention in England, as well as in other parts of the world, they are are exerting their utmost endeavours to prevent it, by disseminating the infection of the small-pox.

But the most dangerous enemies of vaccination are its *pretended friends*; men who *openly* profess to favour the practice, but *secretly* endeavour to undermine it. The public must be surprised to hear, that one individual of this description, who in those writings where his name appears, *extols vaccination*, in his anonymous publications recommends *the general inoculation of the small-pox.*—Such duplicity ought not to pass unnoticed.

There are, it is well known, certain adventurers, who have embarked in that *trading vessel*; and although she is now in a shattered condition, they are still determined to cling to the wreck. They are aware, that their own existence as medical characters is identified with that of the small-pox; and that they must stand or fall together. It is, therefore, unreasonable to expect, that they will ever read a recantation of their errors.

Since the foregoing remarks were sent to the press, another Number of this Journal has appeared, in which Mr. Royston notices Dr. Adams's defence of vaccination, and observes, as Dr. Adams had done before, that no objection had been raised against the practice in any part of the world except England. I have already corrected that error; but had it been otherwise, it is surprising that Mr. Royston did not know what objections have been excited against vaccination in many other countries, particularly in France, Prussia, and America.

I stated on a former occasion, that vaccination had not been propagated through Africa, as Dr. Adams supposed. About that time, the news arrived of the practice being introduced at the Cape of Good Hope, by means of matter brought in a Portuguese ship. This was probably the matter brought by Dr. Balmis, the commissioner sent out on the philanthropic expedition by the King of Spain; as he returned from the Indian Seas in a Portuguese ship, about that period. I am, however, informed by Mrs. Matra, widow of the late Consul, at Tangiers, that Mr. Matra introduced the practice there four years ago, with matter from Gibraltar; but, from want of proper attention in the practitioners, it soon ceased. This circumstance was reported here about the time; but not authenticated till now.

Mr. Royston has also noticed my answer to Mr. Birch; in which, he says, *the warmth*, if not *the asperity*, of my manner, is strikingly characterised. In this laconic nic observation of Mr. Royston, *the warmth*, if not *the asperity*, of *his* manner, is strikingly characterized.

He quotes a passage from a letter of Dr. Pearson, in the Philosophical Magazine for June, 1806, in which it is asserted, that "the publications in favour of vaccination are written, for the most part, with the spirit of animosity, and not of investigation." This is rather unfortunate; since it is well known that many of them, and those none of the mildest, were written by Dr. Pearson himself.

In this passage, quoted from Dr. Pearson by Mr. Royston, with seeming approbation, since he has not corrected the error, we are also told, that neither the friends nor the enemies of vaccination seem to be aware, that vaccination may leave one in a thousand still liable to the small-pox, and yet finally effect the grand object in view, the extermination of the small-pox. This is allowing very little sense indeed to any of the writers on the subject, except Dr. Pearson and Mr. Royston.

I shall prove, that this opinion of Dr. Pearson and Mr. Royston is erroneous. It seems to be intended, in the first instance, as a sarcastic reflection on the Report of the Royal Jennerian Society, published a short time before. It must however, appear evident to every man who reads it, that even that Report contains a refutation of the calumny.

It is entitled, the Report of the Medical Council of the Royal Jennerian Society *for the Extermination of the Small-Pox*, on the subject of vaccine inoculation; and it concludes with a solemn declaration, that in the opinion of the Medical Council, founded on their own experience, and the information which they have collected from others, that mankind have already derived great and incalculable benefit from the discovery of vaccination; and that it is their full belief, *that the sanguine expectations of advantage and security, which have been formed from the inoculation of the cow-pox*, will at length be completely fulfilled. It is well known, that one of the sanguine expectations of advantage and security is, as the very title of the Society implies, *the extermination of the small-pox.*

This Report, though nominally the Report of the Medical Council alone, has been repeatedly sanctioned by the Society at large, and published by their authority. After this, it is rather surprising, that any gentleman who pretends to investigate the subject, should commit such a mistake.

Mr.

Mr. Royston appears to be equally unacquainted with the other circumstances attending the history of vaccination, and the opposition which it has had to encounter. He speaks of Dr. Rowley as if he were the earliest opponent of vaccination; and Dr. Moseley as one who afterwards entered the lists against it, degrading the mental powers which he possesses, becoming the coadjutor of Dr. Rowley in this controversy; and disgracing himself by gross exaggerations, and arts which the vulgar and unlettered understand, and practice, with an adroitness equal to himself.

Mr. Royston sees, or thinks he sees, a greater degree of candour and moderation in the opposition of Mr. Goldson and Mr. Birch, than in that of Dr. Rowley and Dr. Moseley. It appears, however, to me, as well as to others who are conversant with the subject, and have examined the writings of Mr. Goldson and Mr. Birch a little deeper than the surface, that they are equally uncandid; that their moderation is rather pretended than real; and that their hatred of vaccination is full as implacable as that of Dr. Moseley or Dr. Rowley.

Ere I quit this subject, I refer those who doubt the opinion which I have given of the writings of Dr. Pearson, to the publications of Mr. Hicks and Mr. Creaser; and to a lettter from a physician of Bath, in this Journal for 1802; in which Dr. Pearson is accused of hostility to Dr. Jenner, of jealousy, of injustice, and of attempting to rob the discoverer of vaccination of his reward, and of his well-earned fame.

Mr. Royston notices the various defenders of vaccination; one of whom has distinguished himself by his enmity to Dr. Jenner, and another as much by the *moderation* of his talents as of his zeal; and with unparalleled duplicity, been exerting his *influence* to promote the inoculation of the small-pox. Such are the authors whom he holds up to public admiration. Such is the sample of the great work, which we are to expect from him!

---

The next article I shall allude to, in your last Number, is that on burns and scalds. Without pretending to decide a question, agitated by much more experienced practitioners, I beg leave to state one fact, in a patient of my own. A lady covered her foot and ancle with spirits of wine, and then set fire to it, as a remedy for chilblains; but found it extremely difficult to extinguish the flame.

Having often used oil of turpentine in similar cases with apparent advantage, I was much disappointed at the re-

 sult sult in this instance; for it not only aggravated the inflammation in the parts affected, but excited an erysipelas on every part to which it was applied; and even on the other leg, in consequence of its effluvia, which compelled me to discontinue it. I then applied a solution of cerussa acetata, which soon allayed the inflammation; and afterwards healed the sores without difficulty, by the usual means.

---

I now come to the eccentric article, called " An Abridgment of Mr. Marson's Papers on Vaccination; in which he complains of your backwardness in publishing his letters, and asserts that I have always been fortunate in the publication of mine, however prolix. I beg leave, however, to tell Mr. Marson, that a letter may be long without being prolix; and prolix without being long. Besides, a remark of this kind comes with an ill grace from Mr. Marson; who confesses himself guilty of this fault, in a former volume of this Journal; in his dissertation on *close-stools.*

Mr. Marson informs us, that *he* is " one of those long acquainted with variolous inoculation." In this he appears to me to arrogate to himself rather too much; since, as far as I can understand his meaning, he insinuates that those, against whom his arguments are directed, have not been long acquainted with that practice. I have been acquainted with it more than forty years; but do not pretend to know how far that period of probation may be deemed sufficient in the opinion of such a veteran as Mr. Marson.

But Mr. Marson tells us, that he has compared the inoculation of the cow-pock with that of the small-pox: so have I. He has told us, vaccination fell into hands that never inoculated before: so have I.

He tells us, these inexperienced people thought the cow-pock such a mild affection, that a puncture would do the business, and that nothing further was necessary to make vaccination complete. If Mr. Marson's remark means any thing, it means to insinuate, that I am one of those who never inoculated before, and that I am one of those who think a puncture all that is necessary in the inoculation of the cow-pock. As an answer to this insinuation, I refer those who think it worth inquiring into, to what I have written on the subject, in this Journal and elsewhere.

Mr. Marson says, " I do assure you, gentlemen, much is wanting to make vaccination complete; and it is from this imperfection of our knowledge, that disputes and cavils have arisen between THE THEORIST and *the experienced* practitioner." Here again he reminds you, that I am a mere theorist! and when he afterwards tells you, what you very well knew before, that I have the honour of being a Vice-President of the Jennerian Society, he ought to have censured the society, who exalted me to that distinguished situation, from an idea that I had extensive practice. They were even so rash as to suppose, that I understood vaccination almost as well as Mr. Marson.

Mr. Marson says, it is the opinion of Dr. Willan, in his publication, that "the employment of recent lymph seems *no essential requisite*; for the disease is *as often produced by dried as by fluid matter*; though *the former may be more liable to fail.*" This needs no comment.

Mr. Marson's next paragraph is obscure, and almost unintelligible; but, as far as I am able to guess at his meaning, he contends that dry matter is *more likely* to succeed than fluid; an opinion so contrary to that of all other experienced practitioners, that I think it unnecessary to say much about it. I must, however, state, that medical practitioners frequently apply to me for a vaccine patient, from whose arm they may transfer the fluid matter, because they have repeatedly failed with that which is dry.

Mr. Marson says, no particular address is required in performing the operation. No other address is required in performing the operation, than what has often been explained in the various publications on the subject; yet every one who is conversant with the practice knows, that one inoculator succeeds oftener than another. Mr. Marson says, I never impute failures to insusceptibility in the patient. In this he is mistaken; I sometimes impute failures to that cause; but more frequently to some defect in the operation. As to the marks of *a hurried judgment*, as Mr. Marson calls them, I leave to the world to decide, whether they are more conspicuous in my writings or his.

I do not agree with Mr. Marson, that the arm of a healthy subject is in greater danger of violent inflammation, than that of an unhealthy one: but I readily agree with him, that if such a circumstance should occur, neither regimen nor medicine ought to be neglected.

What Mr. Marson says about pulsation is so obscure and unintelligible, if not contradictory, that I shall leave it to himself to explain it. When he talks about my prejudices, and modestly hints that my opinions are erroneous, from a want of practical attention; I beg leave to say, that my practice and information on the subject are far more extensive; and my attention at least equal to his. He recommends to me the plan of treating ten patients with diet and regimen and ten without. I thank him for his advice; but having seen sufficient of the effects of such a plan in the practice of others, to convince me of its inutility, and of its injurious effects, I must be excused from making the same experiment.

I am, &c.

JOHN RING.

*New-Street, Hanover-Square.*

